# Transcription–translation coupling: Recent advances and future perspectives

**DOI:** 10.1111/mmi.15076

**Published:** 2023-05-15

**Authors:** Jason Woodgate, Nikolay Zenkin

**Affiliations:** ^1^ Centre for Bacterial Cell Biology, Biosciences Institute, Faculty of Medical Sciences Newcastle University Newcastle Upon Tyne UK

**Keywords:** bacterial gene expression, coupling of transcription and translation, CryoEM, expressome, molecular machines, transcription, translation

## Abstract

The flow of genetic information from the chromosome to protein in all living organisms consists of two steps: (1) copying information coded in DNA into an mRNA intermediate via *transcription* by RNA polymerase, followed by (2) *translation* of this mRNA into a polypeptide by the ribosome. Unlike eukaryotes, where transcription and translation are separated by a nuclear envelope, in bacterial cells, these two processes occur within the same compartment. This means that a pioneering ribosome starts translation on nascent mRNA that is still being actively transcribed by RNA polymerase. This tethering via mRNA is referred to as ‘coupling’ of transcription and translation (CTT). CTT raises many questions regarding physical interactions and potential mutual regulation between these large (ribosome is ~2.5 MDa and RNA polymerase is 0.5 MDa) and powerful molecular machines. Accordingly, we will discuss some recently discovered structural and functional aspects of CTT.

## Graphical abstract

We present a brief review and perspective of the current understandings and future directions in investigations of the coupling of transcription and translation in bacteria. We discuss recent biochemical and structural data to suggest potential focus points for those in the field.

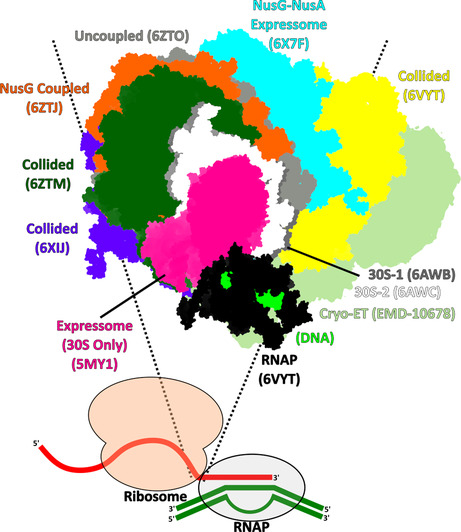

## 1 INTRODUCTION

To start transcription, RNA Polymerase (RNAP) locally melts the DNA duplex, forming a transcription bubble, to provide a single‐stranded region that guides polymerisation of a complementary mRNA strand. The growing mRNA forms a ~9 nucleotide‐long RNA:DNA hybrid within the transcription bubble of the elongation complex (EC). During mRNA synthesis and translocation of the EC, the 5′ end of mRNA disengages from the template DNA and exits the EC, thus keeping the size of transcription bubble and RNA:DNA hybrid constant throughout transcription elongation, reviewed in Nudler (2009). The 5′ section of mRNA that has left the EC soon after the start of transcription, therefore becomes available for translation to start. To begin translation, the ribosome assembles upon the nascent mRNA's ribosome binding site (RBS) aided by initiation factors 1–3 and GTP. The start codon AUG is recognised by the initiator fMet‐tRNA^fMet^. Translation elongation uses aminoacylated tRNAs that, by matching complementary trinucleotide codons of mRNA, determine the sequence of the synthesised protein, that is, decoding of the genetic information. Translation elongation factors EF‐Tu and EF‐G use the energy of GTP hydrolysis to ensure the binding of correct and charged tRNA to the ribosome and ribosome translocation after peptide bond formation, respectively, reviewed in Rodnina (2018).

Considering that coupling involves tethering via mRNA and possible physical contact between machines, it can be expected for both machineries to influence each other directly and, potentially, other processes on DNA through the coupled EC. The ribosome is a powerful molecular machine utilising hydrolysis of two high‐energy diphosphate bonds and peptide bond formation to accelerate and bias directionality of the molecular ratchet translocation powered by Brownian motion (Finkelstein et al., 2018). Comparatively, the EC employs hydrolysis of a single diphosphate bond during nucleotide monophosphate incorporation into nascent RNA that biases directionality of translocation along the DNA, which is powered solely by Brownian motion (Bar‐Nahum et al., 2005; Belogurov & Artsimovitch, 2019). At the same time, however, the EC's extensive grip upon an RNA–DNA hybrid and DNA of the transcription bubble ensures unusual stability of the EC. Consequently, it is not immediately clear how each machine will fare upon physical interaction.

**FIGURE 1:**
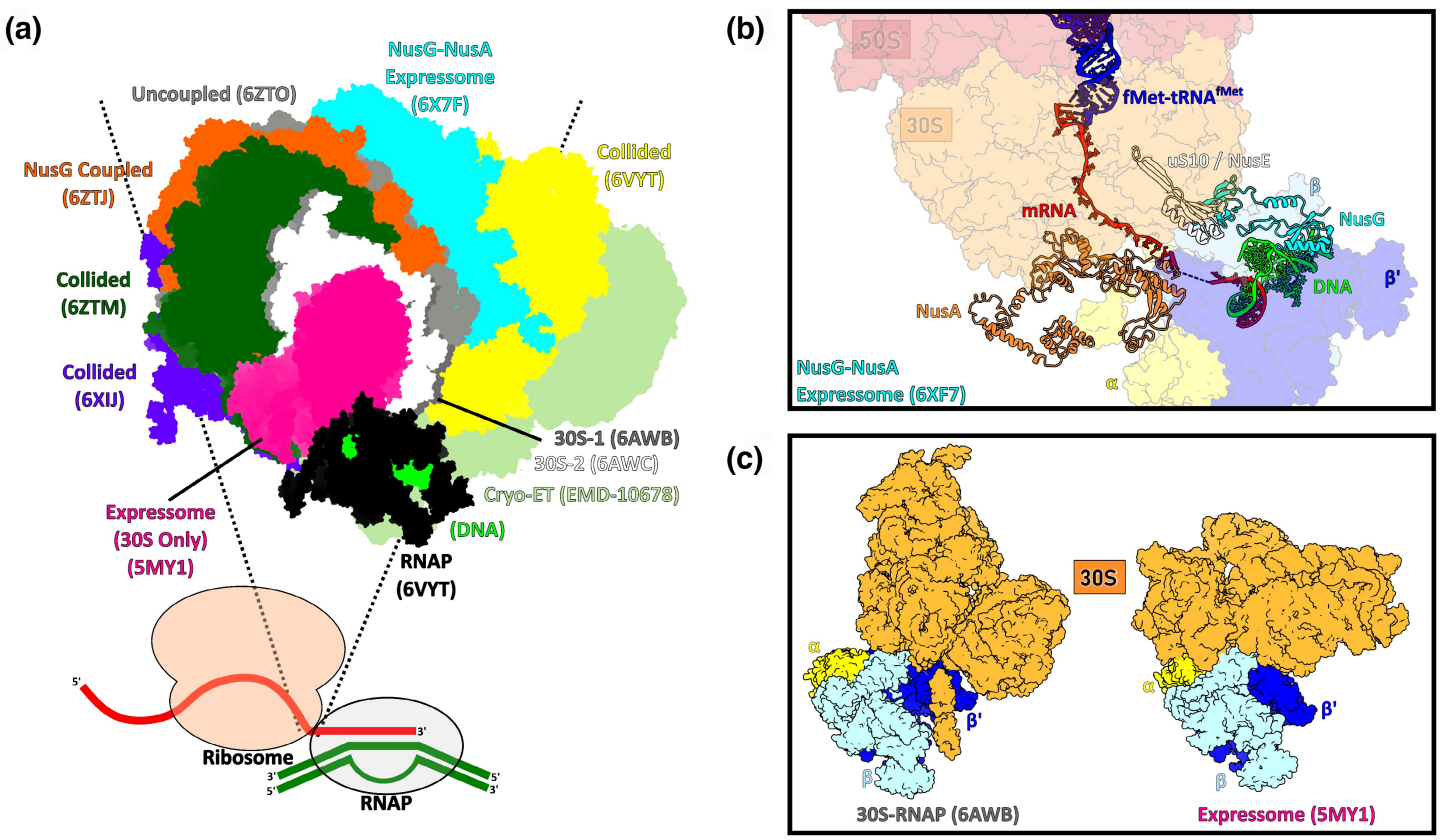
Structural Investigations of CTT. (a) Comparison between complexes of ECs and ribosome. Complexes are aligned by RNAPs, and ribosome position and orientation of each model overlain. Below is a schematic representation of the coupled complex, with dotted lines highlighting overlap axis. The ‘uncoupled’ model describes CTT with intermachine mRNA lengths of >38 nucleotides and without factors. PDB IDs are shown next to the structures: 6X7F, 6VYT, 6XIJ (Wang et al., 2020); 6ZTO, 6ZTM, 6ZTJ (Webster et al., 2020); 6AWB, 6AWC (Demo et al., 2017); 5MY1—available PDB deposition is of EC and 30S subunit only (Kohler et al., 2017). EMID map ID provided for Cryo‐ET map of *M. pneumoniae* in‐cell expressome (O'Reilly et al., 2020). (b) Structural model of NusG–NusA bridging within factor‐assisted expressome. (c) Comparison between orientation of 30S ribosome subunit and RNAP within the first expressome model (5MY1) and a free complex of RNAP and 30S subunit (6AWB).

The CTT story as we know it began in the 1970s when we were given the first visual glimpse into bacterial gene expression, through the electron micrographs of so‐called Miller spreads (Miller et al., 1970). These depict RNAP transcribing on DNA in lysed cells, with 70S ribosomes sitting upon the nascent mRNA behind it. Such a powerful image has set the tone for bacterial cellular and genetic biology, with new assumptions based on knowledge of this close‐coupled nature of gene expression in bacteria. Since then, we have learned a great deal regarding the nature and importance of CTT, and specifically, within the last 8–10 years we have seen an intense increase in CTT investigations through cellular biology, biochemistry and structural biology. Within this perspective piece, we briefly discuss a handful of the interesting models generated from those investigations of CTT, including a comparison of structural models (Figure 1a), limitations of these models and questions that remain unanswered.

## 2 THE CLASSICS OF CTT

The two fundamental processes of gene expression must be correctly regulated and sufficiently processive, free from deleterious collisions and traffic jams. It has been documented that the overall rates of transcription and translation are matched during cell growth (Proshkin et al., 2010) of around ~20–100 nucleotides s^−1^ and ~14–20 amino acids s^−1^ (Iyer et al., 2018; Proshkin et al., 2010), respectively. While this average transcription rate has also been reported at ~90 nucleotides s^−1^ on rRNA operons (Condon et al., 1993; Vogel & Jensen, 1994), the EC has been observed transcribing at 250–400 nucleotides s^−1^ upon these untranslated genes (Dennis et al., 2009). So, it is reasonable to suggest that the matching of the rates of the two machines during coupling on protein‐coding sequences may be a specific mechanism, evolved for efficient bacterial gene expression. In this scenario, the ribosome can be understood as a directly acting, bona fide, transcriptional regulator, which assists otherwise slow transcription on protein‐coding sequences. For example, the leading ribosome can step in to restore processivity of a paused EC, likely through physical pushing (Proshkin et al., 2010; Stevenson‐Jones et al., 2020). More recent biochemical and single‐molecule analyses showed that a coupled ribosome can increase transcription processivity, though at the expense of fidelity of RNA synthesis, through mechanical and allosteric means (Wee et al., 2023). These findings are corroborated by in vivo observations that the ribosome appears to assist the progression of EC inhibited by carbon starvation, while average rates of transcription are reduced when translation is inhibited under nitrogen‐limiting conditions (Iyer et al., 2018). It should be noted, however, that in the latter case, the slowing of translation by nutrient deprivation may activate stringent response that leads to the production of the global small molecule regulator (p)ppGpp which, alongside the transcription factor DksA, can reduce transcription elongation rates (Roghanian et al., 2015; Vogel et al., 1992; Zhu et al., 2019). Below, we will focus on crosstalk between the coupled EC and the leading ribosome and will not discuss the indirect mechanisms such as the stringent response.

The timing of coupling and changes in the gap between leading ribosome and the EC may play an important role in the fate of the expression of particular genes. The most pertinent examples of gene expression regulation through CTT are attenuation (Chan & Landick, 1993; Yanofsky, 1981) and polarity (Adhya & Gottesman, 1978; Roberts, 1969). The best studied examples of attenuation are the *trp* (Landick et al., 1985) and *his* (Chan & Landick, 1993) operons, responsible for the synthesis of Tryptophan (Trp) and Histidine, respectively. Transcription of these genes is controlled by the availability of the corresponding amino acids in the growth media, which is sensed by the coupled translation. Attenuation regulation is based on the overlapping signals for transcription termination and ribosome pausing in the absence of a particular amino acid. For example, when Trp is in abundance, coupled ribosome translates a short open reading frame in the initially transcribed region of the *trp* operon without pausing on two Trp codons. This allows the nascent mRNA to fold into a hairpin and terminate the EC before it has transcribed the whole *trp* operon, as there is no need for the production of these enzymes in such conditions. In conditions of low Trp, the ribosome pauses within this short open reading frame at Trp codons. This leads to an alternative antiterminator secondary structure of the nascent mRNA behind the transcribing EC, which allows transcription of the whole operon (Landick et al., 1985).

In the case of transcription polarity, the coupled ribosome helps the progression of an EC that synthesises mRNAs containing C‐rich and G‐sparse Rho Utilisation (*rut*) sequences. These sequences in transcripts enable the binding of Rho, a homohexameric RNA translocase, that drives itself along the transcript powered by ATP hydrolysis, reviewed in (Richardson, 2002). Upon reaching the rear end of the active EC, Rho is thought to terminate transcription through allosteric manipulation of the EC (Epshtein et al., 2010; Song et al., 2022). The transcription elongation factor NusG acts as a recruitment aid for Rho, localising the translocase to active ECs and increases the chances of Rho recruitment to mRNA. NusG binds via its N‐terminal domain (NTD) to RNAP (Mooney et al., 2009) and reaches out with its C‐terminal domain (CTD) to bind Rho (Washburn et al., 2020). However, at the same time, the ribosomal NusE (protein S10 of 30S subunit) also competes for the CTD of NusG (Burmann et al., 2010; Saxena et al., 2018). The overall rate of translation itself determines the gap between leading ribosome and the EC, that is, how much of the mRNA is exposed for Rho binding. If the ribosome has slowed or stopped, *rut* sites on the mRNA become exposed, while NusG bound to the EC is not in contact with NusE and is available for interaction with Rho. This allows Rho factor to bind and drive transcription termination, resulting in the phenomenon of polarity, when an uncoupled EC cannot reach the end of long genes or operons, being terminated by Rho on its way. In contrast, if the translation rate matches that of transcription, the approach of Rho to the EC is blocked by the ribosome, while NusG cannot assist in Rho recruitment, having been sequestered by NusE of the ribosome (Adhya & Gottesman, 1978; Burmann et al., 2010; Saxena et al., 2018).

The NusG‐paralog RfaH is also implicated within CTT (Zuber et al., 2018). The lower copy number RfaH shares a binding site with NusG and also reaches across from the EC to form a bridge with the ribosomal NusE. However, RfaH acts specifically on EC paused at a sequence known as the operon polarity sequence (*ops*) (Bailey et al., 1997). Such specificity is partly caused by the presence of small hairpin within the nontemplate DNA strand of the transcription bubble of the *ops*‐paused EC (Bailey et al., 1997; Zuber et al., 2018). RfaH is also an interesting example in the world of proteins, undergoing a unique CTD transformation from autoinhibitory α‐helix to a NusG‐like β‐barrel upon binding to this small nontemplate DNA hairpin within the EC at the *ops* pause, thanks to its metastable folding landscape (Belogurov et al., 2007; Burmann et al., 2012). Thus, differing from NusG, RfaH is expected to act specifically only upon the ribosome coupled to EC paused at the *ops* DNA sequence. RfaH‐mediated ribosome recruitment is required to limit transcriptional pausing and polarity at some virulence genes (Hustmyer et al., 2022), indicating that the state of CTT could be of importance in bacterial infections.

## 3 ATTEMPTS IN STRUCTURAL UNDERSTANDING OF CTT

Close relations between a leading translating ribosome and transcribing RNAP have led to the suggestion that they may form a physical complex, referred to as the ‘expressome’, that is, a molecular super‐assembly performing both steps of gene expression. This initiated a number of structural studies into the matter.

Within a recent study, NusG, along with another ubiquitous bacterial factor NusA, was suggested to participate in the formation of one of the types of expressomes (Wang et al., 2020). Within such a factor‐assisted expressome, the ribosome and EC are depicted as physically bridged by NusA and NusG (Figure 1b), restricting the freedom of motion of each machine relative to one another, compared with CTT without the NusA–NusG frame (see below). The assembly was shown to form a new intermachine mRNA channel from the ribosomal S3 and S1, EC flap and zinc finger, and the Nus factors, protecting and stabilising the mRNA. The proposed distance between ribosome and RNAP in such a complex was 41–47 nucleotides (here and after, the distance between machines will be referred to as number of nucleotides of mRNA between active centres of ribosome and RNAP). Such an expressome was not seen when using mRNAs of shorter intermachine distances. Though not tested on longer mRNAs, the authors suggest up to 50 nucleotides of mRNA are possible to be accommodated between active centres of the machines in the factor‐assisted expressome. A separate study corroborated the existence of the NusG‐expressome, at similar distances between the machines, albeit without NusA (Webster et al., 2020). Modelling using the factor‐assisted expressome indicated that the ECs orientation relative to the ribosome would allow for mRNA looping, and accordingly, the EC is positioned to retain the responsiveness to external factors and mRNA‐driven signals for transcription elongation (Wang et al., 2020). The authors also suggest that the factor‐assisted expressome would likely be the de facto state for CTT under common elongation circumstances.

The first study that reported expressome, was, in fact, obtained without NusA/NusG factors, and was formed by colliding elongating ribosome into the EC (Kohler et al., 2017; later it was referred to as ‘collided’). Several ‘collided’ assemblies were also investigated later using mRNA of between 26 and 34 nucleotides between artificially stalled ribosome and EC (Wang et al., 2020; Webster et al., 2020) and showed slightly different EC‐ribosome orientations from one another and the first ‘collided’ expressome model (Kohler et al., 2017; Figure 1a). However, a variability analysis that statistically describes the relative freedom of the EC and the ribosome within CTT through the generation of ~18,000 hypothetical models of a CTT structure (Webster et al., 2020) suggests that these ‘collided’ structures are most likely just a handful of many conformations in time and space for the EC and ribosome. At larger distances (>38 nucleotides) between machineries, and in the absence of factors, the EC has much greater freedom relative to the ribosome. This suggests that it could be just the length of the mRNA that limits the possible freedoms between the EC and the ribosome (Webster et al., 2020), rather than a formation of bona fide complex between the machines. The ribosome orientation relative to the EC within the factor‐assisted expressome differs substantially from ‘collided’ expressomes (Wang et al., 2020; Webster et al., 2020; Figure 1a). The ‘collided’ structures also indicate an incompatibility with NusG/NusA binding and a lack of capacity for mRNA looping compared with the factor‐assisted structure (Wang et al., 2020; Webster et al., 2020). Modelling indicates that, in the ‘collided’ expressome, the EC can no longer respond to cues dependent on mRNA such as pause and termination hairpins (Wang et al., 2020). The ribosome in the ‘collided’ expressome was also predicted to be incapable of completing a successful translocation cycle, due to steric hindrance of RNAP, blocking the conformational changes of 30S subunit required for the ratcheting during the translation elongation cycle (Wang et al., 2020; see also next section).

The aforementioned models provided interesting insights into the relations between ribosome and EC. However, the existence of expressome was proposed solely based on these CryoEM structures and was not known a priori. A proportion of these structures were obtained after chemical cross‐linking of the components prior to the CryoEM grid preparation (Kohler et al., 2017; Webster et al., 2020). This procedure may enrich for a minor fraction of actual repertoire of complexes between ribosome and the EC, stabilise intermachine relative motion, or lead to the formation of artificial assembles. Furthermore, the functional relevance of the assemblies analysed by CryoEM was not verified. For example, in the study by Wang et al., the translation elongation complex was mimicked by mixing ribosomes with just deaminoacylated tRNA^fMet^ (Wang et al., 2020). Another study by Webster et al. for the same purpose used more natural tRNAs, that is, aminoacylated and formylated initiator fMet‐tRNA^fMet^ and aminoacylated Phe‐tRNA^Phe^, though without translation elongation factors (Webster et al., 2020). Whether these more ‘initiation‐like’ ribosomes can represent the translation elongation complexes within active CTT is yet to be demonstrated. It is also worth noting that the ribosomes in some of the ‘collided’ complexes were forced to bind mRNA at distances from the EC, which previously were noted to preclude initiation in vitro, for example, 26–30 nucleotides between active sites (Castro‐Roa & Zenkin, 2012, 2015). The study by Kohler et al. that was first to model the expressome structure used a ribosome elongating towards the EC in the presence of all aminoacyl‐tRNAs (Kohler et al., 2017). While being a more natural set‐up for possible expressome formation, the states of ribosome and EC after the reaction were not established; for example, whether ribosome reached the EC (see below), and what happened to RNAP upon such collision is not known. As can be seen from Figure 1a, the relative orientation of ribosome and EC in this expressome complex was different to the other structures discussed above highlighting uncertainty in how some of the artificial assemblies reflect the real active CTT. Furthermore, in all mentioned studies ECs were assembled with bespoke nucleic acid scaffolds with no verification of the states of the ECs or their activities in the coupled systems. Such a static nature of the assemblies means we still do not know how each machine can accommodate/adopt one another during *active* transcription and translation.

The most recent structure/function study highlighted one functional interaction within CTT (Wee et al., 2023). The authors describe the ribosome acting allosterically to inhibit a swivelling conformation change in RNAP that occurs during EC pausing and backtracking (Abdelkareem et al., 2019; Kang et al., 2018). In this way, the ribosome biases the EC for processivity, though at a cost of fidelity. Interestingly, while the authors used all required factors and correct tRNAs for the complex assembly and CryoEM structure determination, they found low efficiency of translation elongation causing ribosomes to pause at earlier codons. This resulted in higher variability in CryoEM maps, than the static assemblies seen before. The authors had to remove 80% of particles, leaving those with the least variation. The resulting structure resembled the ‘collided’ expressome. However, the causes of variability among individual particles and their functional significance remain unclear.

Interestingly, one study reported complexes between free small ribosomal subunit 30S and free RNAP (in the absence of mRNA or DNA; Demo et al., 2017). The authors, however, suggested that it was not expressome per se but a complex that may play role in initiation of translation. Translation initiation starts with 30S subunit binding to ribosomal binding site on mRNA, that may be accelerated by association of 30S with RNAP that has just started synthesis of an mRNA. Notably, the orientation of the 30S relative to RNAP in this complex was quite different from any of the expressomes discussed above (Figure 1a–c).

## 4 CONTROVERSIES BETWEEN STRUCTURAL AND FUNCTIONAL FINDINGS

The minimal, or contact, distance between the two coupled elongating machines on mRNA was found biochemically to be 25–26 nucleotides (Stevenson‐Jones et al., 2020). At this distance, the ribosome was shown to be able to physically push a backtracked EC forward along the DNA, providing a route to reversal of backtracking (Stevenson‐Jones et al., 2020), which may be important for genomic stability through prevention of conflicts of transcription with other molecular processes (Dutta et al., 2011; Gamba & Zenkin, 2018; Yuzenkova et al., 2014). An EC rescued by the leading ribosome from backtracking can continue transcription away from this ribosome without delay, even if the ribosome is artificially stopped (Stevenson‐Jones et al., 2020). This suggests that, if an expressome exists, the looping of mRNA between machines must be very flexible—shortening to ‘zero’ (25 nucleotides between active centres) when the EC and ribosome come into contact, and then extending indefinitely—without interfering with functions of either of the machines. This, however, raises the question of the biological sense of the existence of a ‘permanent’ expressome, in which the EC and ribosome are connected only structurally, but not functionally. Furthermore, the fact that the ribosome comes to contact distance with the EC, and actively pushes it while continuing synthesis of nascent peptide (Stevenson‐Jones et al., 2020; Wee et al., 2023), argues against the structural prediction that leading ribosome ratcheting is blocked in the vicinity (26–34 nucleotides) of the coupled EC. As a consequence, the functionality of the structure of collided ribosome and EC, where the ribosome is inactivated, is yet to be understood. Interestingly, no effects of coupling factors NusA and NusG have been seen in vitro in a reconstituted coupled system so far (Stevenson‐Jones et al., 2020) and, whether there is a functional significance in their absence or presence in different expressome models, remains a question.

The current models describing bacterial CTT are built from data primarily based on the most common lab model organism, Gram‐negative *Escherichia coli*. There are, however, examples that argue against the idea of transcription and translation coupling being a constant and required presence in all bacteria. In the case of *Bacillus subtilis*, it was recently shown that an active EC, more often than not, escapes from the trailing ribosome, suggesting that transcription and translation might be functionally uncoupled (Johnson et al., 2020). Phylogenetic analysis, using the short distance between stop codons and transcription terminator hairpins as a conservative signature for the lack of kinetic coupling, predicted that uncoupling of transcription and translation could be common for all Firmicutes (Johnson et al., 2020). These findings are also supported by the evidence of reduced transcriptional attenuation by coupled ribosome in *B. subtilis*. Instead, there is an increased abundance of transcription‐controlling riboswitches (Babitzke & Yanofsky, 1993) that would not be compatible with CTT. Also a minimal role of Rho‐driven transcription termination in *B. subtilis* suggests that protection of the EC by the coupled translation is no longer necessary (Ingham et al., 1999). Furthermore, the NusG, coupling factor in *E. coli*, plays a different role in transcription for *B. subtilis*, mainly to increase pausing (Yakhnin et al., 2016). Together, these data suggest that not all bacteria may require tight transcription–translation coupling or have similar mechanisms of coupling, and generalisation of finding on model CTT to all bacteria may not be valid.

All these interesting controversies indicate that we are barely scratching the tip of the iceberg of the mechanistic aspects of the coupling of transcription and translation, and further biochemical, microbiological and structural studies are needed before relations between two steps of gene expression in bacteria are understood.

## 5 CONCLUDING REMARKS

How structurally observed interactions of the mimics of the elongating ribosome with the EC represent the actual expressome, and if the expressome, as a structure‐functional complex between ribosome and EC, exists, is yet to be understood. Accordingly, some of the aforementioned structures are very different in relative orientation of RNAP and ribosome from the others (Figure 1a), that is, some of them potentially being derived from assemblies that may not be representative of states that predominate naturally in the cell. It is, therefore, likely that these structures represent snapshots of the multiple functional or nonfunctional interactions between the two machineries and assuming any likelihood of adoption of these assemblies during natural CTT at this stage could be misleading.

It is possible that the observed contact interfaces between EC and ribosome could have evolved to tolerate one another, thus allowing for efficient independent function, though enabling some situation‐specific interactions. For example, we might expect that 30S‐RNAP complex will assist colocalisation of machines to increase effective concentrations for the correctly timed and regulated translation initiation process upon mRNA (Demo et al., 2017), but not represent the majority of CTT in the cell. We also note that a recent cryo‐electron tomography (Cryo‐ET) study of *M. pneumoniae* cells showed that some form of expressome does form in vivo, at least temporarily, in response to a specific stimulus (in response to antibiotic challenge; O'Reilly et al., 2020). RNAP orientation relative to the ribosome within this in‐cell‐expressome aligns closer to the ‘collided’ *E. coli* in vitro expressome than that of the factor‐assisted expressome (Figure 1a). However, how critical is the possible formation of various expressomes for different bacteria is yet to be understood.

In this perspective, we focussed on the advances in understanding the mechanistic relations between ribosome and RNAP. There are however multiple processes that may depend on the crosstalk between the two machineries. DNA repair relies heavily on EC recognising the lesions in DNA (transcription‐coupled repair), suggesting that this activity can be affected by coupled ribosomes. Other processes taking place on the DNA, such as replication and associated transcription–replication conflicts, may also be affected by CTT through the monitoring of stalled ECs. CTT may be involved in organisation of the chromosome due to phase separation of cytoplasm containing ribosomes and chromosome containing ECs. Membrane proteins need to be inserted into membrane co‐translationally and, accordingly, co‐transcriptionally, which raises the question of the CTT‐dependent rearrangement of the gene location towards the membrane (a phenomenon called transertion; Kaval et al., 2023). These and many other questions about the role of coupling of transcription and translation in life of various bacteria remain to be answered, and exciting recent advances will serve as a springboard to do that.

## AUTHOR CONTRIBUTIONS

Jason Woodgate developed concept and wrote the review, and Nikolay Zenkin participated in writing the review.

## CONFLICT OF INTEREST STATEMENT

The authors declare no conflicts of interest.

## ETHICS STATEMENT

This review does not involve human subjects, patient medical records or animals.

## Data Availability

Data sharing is not applicable to this article as no new data were created or analyzed in this study.
